# Valiltramiprosate Effects on Microstructural Integrity of Grey and White Matter in APOE4/4 Homozygotes with Early AD and their Correlations to Clinical Outcomes: MRI Mean Diffusivity Results from the 78‐Week APOLLOE4 Phase 3 Trial

**DOI:** 10.1002/alz70861_108716

**Published:** 2025-12-23

**Authors:** Earvin Liang, Susan Abushakra, P. Murali Doraiswamy, Duygu Tosun, Marwan N. Sabbagh, Anton P. Porsteinsson, David Watson, Aidan Power, Sharon Cohen, Emer MacSweeney, Chris Conklin, Madhura Ingalhalikar, John A. Hey, Patrick Kesslak, Susan Flint, Arthur Rosenthal, Adem Albayrak, Martin Tolar

**Affiliations:** ^1^ Alzheon Inc., Framingham, MA USA; ^2^ Alzheon, Inc., Framingham, MA USA; ^3^ Neurocognitive Disorders Program, Department of Psychiatry, Duke University School of Medicine, Durham, NC USA; ^4^ Center for Imaging of Neurodegenerative Diseases, San Francisco Veterans Affairs Medical Center, San Francisco, CA USA; ^5^ Barrow Neurological Institute, Phoenix, AZ USA; ^6^ University of Rochester School of Medicine and Dentistry, Rochester, NY USA; ^7^ Alzheimer's Research and Treatment Center, Wellington, FL USA; ^8^ Toronto Memory Program, toronto, ON Canada; ^9^ Re‐Cognition Health, London, Greater London UK; ^10^ Clario, Inc., Philadelphia, PA USA; ^11^ Alzheon, Framingham, MA USA

## Abstract

**Background:**

Valiltramiprosate/ALZ‐801, an oral inhibitor of amyloid oligomer formation, was evaluated in a Phase 3 trial in APOE4/4 subjects with Early AD. While the study did not meet its primary clinical endpoint, volumetric MRI (vMRI) showed significant brain atrophy slowing. The pre‐specified MCI subgroup showed positive effects on both clinical *and* vMRI outcomes. Amyloid oligomers are hypothesized to cause synaptic dysfunction and axonal damage, leading to microstructural changes and volume loss. The effects of valiltramiprosate on microstructural tissue changes in grey/white matter (GM, WM) were investigated using mean water diffusivity (MD) on diffusion MRI (dMRI‐MD).

**Method:**

This study enrolled 325 APOE4/4 homozygotes (162 placebo, 163 valiltramirposate) stratified by MCI/Mild AD. The ADAS‐Cog13/CDR‐SB were primary/key secondary outcomes; hippocampal volume and cortical thickness (HV, CT) were main/secondary vMRI outcomes, respectively. dMRI from 1.5/3 Tesla scanners were centrally analyzed by Clario Inc. (lower MD indicates tissue preservation). MMRM was primary analysis; Pearson’s correlations were conducted.

**Result:**

The clinical/vMRI datasets included 320/290 subjects respectively; dMRI included 206 subjects (84 MCI, 122 Mild AD). In the overall population, valiltramiprosate effects on MD compared to placebo showed positive trends (*p* <0.1) on cingulate/occipital cortex and caudate/striatum, and significant WM effects (corpus callosum genu [GCC] *p* <0.001; fornix *p* =0.007). Mild AD showed positive trends on occipital cortex and significant WM effects (GCC *p* =0.005; fornix *p* =0.019). MCI showed significant effects in cingulate (*p* =0.031) and WM (GCC/whole CC: *p* =0.003/0.013) and fornix (*p* =0.032). In MCI active arm, dMRI effects correlated significantly with clinical and volumetric effects. Frontal cortex MD correlated significantly with ADAS‐Cog13/CDR‐SB (*p* < 0.05) and HV and CT (both *p* =0.002). WM‐GCC diffusivity correlations to HV (*p* =0.03) and CT were significant (*p* =0.006).

**Conclusion:**

Consistent with valiltramiprosate’s effects on brain volumes, its effects on microstructural integrity were most prominent in the MCI subgroup that showed lower diffusivity in the cingulate cortex and major hippocampal outflow tracts. This microstructural preservation correlated significantly with preservation of HV/CT and with clinical benefits. These findings support valiltramiprosate’s proposed mechanism: inhibiting oligomer formation may directly protect synapses/axons from damage, thus slowing neurodegeneration at a microstructural level and contributing to macrostructural preservation of brain volumes and clinical benefit.

